# Congenital heart disease presentations in the 15q11.2 microdeletion syndrome

**DOI:** 10.3389/fgene.2025.1535732

**Published:** 2025-03-19

**Authors:** Claudia-Ioana Fifirig, Sabu Abraham, Bernard Keavney, Kathryn E. Hentges

**Affiliations:** ^1^ BHF Manchester Centre of Research Excellence, Division of Evolution, Infection and Genomics, School of Biological Sciences, Faculty of Biology, Medicine and Health, Manchester Academic Health Sciences Centre, University of Manchester, Manchester, United Kingdom; ^2^ BHF Manchester Centre of Research Excellence, Division of Cardiovascular Sciences, School of Medical Sciences, Faculty of Biology Medicine and Health, University of Manchester, Manchester, United Kingdom; ^3^ Manchester Heart Centre, Manchester University NHS Foundation Trust, Manchester Academic Health Science Centre, Manchester, United Kingdom

**Keywords:** congenital heart disease, genetics, development, BP1-BP2/15q11.2 deletion syndrome, copy number variant, phenotypic heterogeneity, low penetrance

## Abstract

Congenital heart disease (CHD) is the most common type of birth defect and results from anomalies in the cardiogenesis process. There are multiple genetic mechanisms contributing to CHD, including copy number variants (CNVs). One such CNV is the 15q11.2 (BP1-BP2) microdeletion, which contains four evolutionarily conserved genes: *NIPA1*, *NIPA2*, *CYFIP1,* and *TUBGCP5*. The deletion causes a syndrome which includes developmental delays and multiple anatomical malformations including CHD. The link between the 15q11.2 (BP1-BP2) microdeletion and CHD has been previously described in the literature but not explored in terms of mechanistic investigations. The characteristics of the BP1-BP2 deletion also prove challenging in the context of genetic counselling. Here we discuss the 15q11.2 (BP1-BP2) microdeletion syndrome with a focus on CHD.

## Introduction

CHD is defined as structural abnormalities of the heart and/or the great vessels which are present at birth. CHD is the most common type of birth defect affecting between 0.8% and 1.2% of live births worldwide. CHD can appear as isolated defects, be accompanied by extra-cardiac abnormalities or be an aspect of known genetic syndromes (Bouma and Mulder, 2017; Miller et al., 2011; Zaidi and Brueckner, 2017). Advances in diagnosis and treatment have greatly reduced the mortality rate of CHD, but patients still suffer severe detriments to their quality of life as they are at a greater risk of adverse cardiac events and for developing secondary conditions (Zaidi and Brueckner, 2017; Wu et al., 2020; Diller et al., 2015; Liu et al., 2019; Marelli et al., 2014).

**FIGURE 1:**
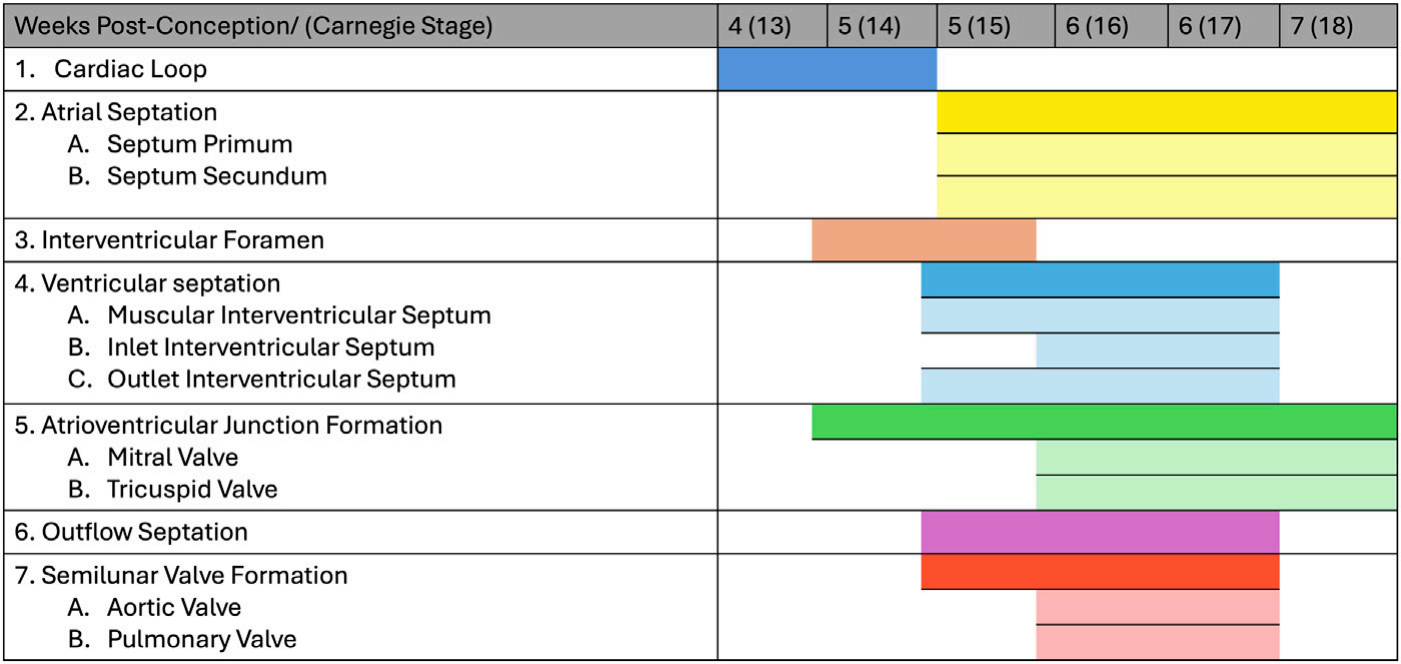
Human heart development stages. The figure shows the development of the human heart by weeks post-conception and corresponding Carnegie stage.

Heart development is a complex process that has been heavily investigated and described in numerous animal models. The heart is the first organ that forms and functions in the developing embryo and its developmental process follows a similar general pattern in all vertebrates from fish to humans. The main stages of the cardiogenesis process are shown in detail in Figure 1. The process of cardiogenesis can be perturbed at various stages causing defects which, unless lethal, will be present at birth (Kaufman and Bard, 1999; Savolainen et al., 2009; Papaioannou and Behringer, 2005).

Despite progress made with treatment, the genetic causes underpinning CHDs remain largely unknown. Up to 80% of isolated CHD cases remains without a genetic diagnosis (Blue et al., 2017), and only ∼15% of overall CHD cases are solved. Due to high heterogeneity, variable penetrance and associated developmental disorders, identifying the causes of CHD proves a difficult undertaking. However, it has been shown that genetics and genetic disorders play a key role in CHD (Bouma and Mulder, 2017; Miller et al., 2011). It is estimated that 13% of CHD cases can be attributed to aneuploidy, 10% to copy number variants (CNVs), 10% to *de novo* single nucleotide variants, 10% to environmental causes and 1% to inherited genetic variants (Zaidi and Brueckner, 2017). Genomic CNVs result from the deletion or duplication of a continuous DNA fragment and have been associated with CHD both in a syndromic and non-syndromic fashion. One such CNV is the 15q11.2 (BP1-BP2) microdeletion (Soemedi et al., 2012) which is the topic of this review.

### The 15q11.2 (BP1-BP2) region

**FIGURE 2:**
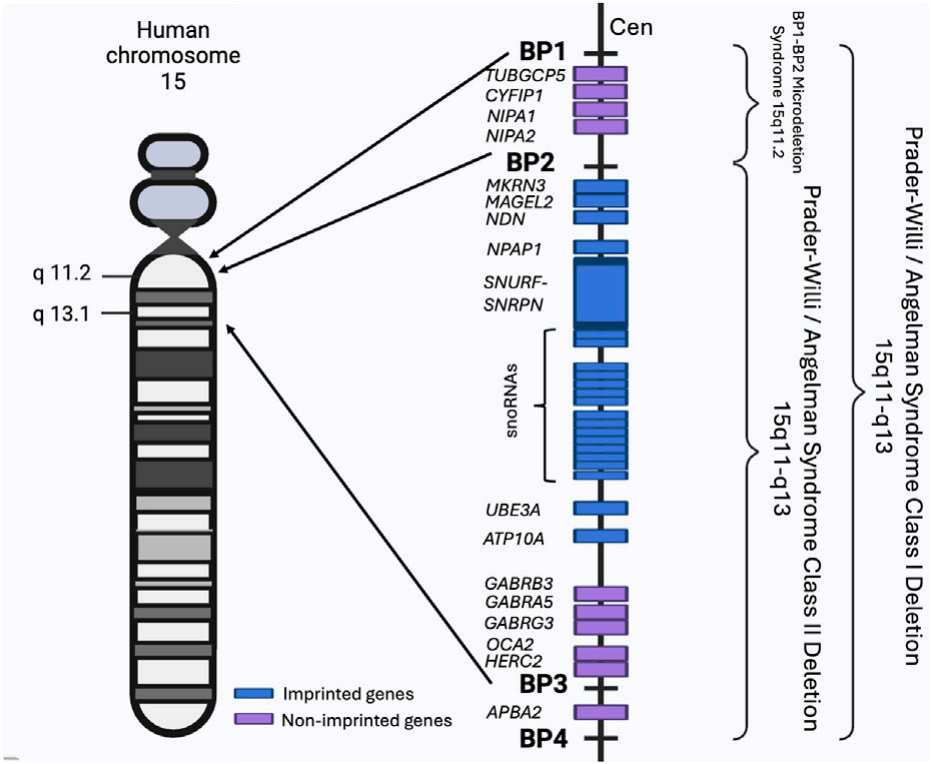
The BP1-BP4 regions and associated syndromes. The schematic shows the BP1-BP4 regions on the human chromosome 15, their component genes (not to scale) and all known associated syndromes. Chromosome regions are numbered and shown on the side of the chromosome. Made using BioRender (https://BioRender.com/e31r278).

In the proximal region of its long arm, human chromosome 15 contains five low-copy repeats clusters, known as breakpoints 1–5 (BP1-5). These clusters are associated with non-allelic homologous recombination (NAHR) which is among the commonest mechanisms underpinning CNVs. The BP1-BP5 segment includes the Prader-Willi Syndrome (PWS) and the Angelman Syndrome (AS) imprinted regions as shown in Figure 2. These two syndromes differ in phenotype and are caused by the deletion of the 15q11-q13 region in either the paternal chromosome for PWS or the maternal chromosome for AS. The deletions include either BP1-BP3 (class I) or BP2-BP3 (class II). Many reports indicate that patients with class I deletions have more severe phenotypes than class II patients, which highlights the importance of the BP1-BP2 region and its component genes (Mohan et al., 2019).

Individuals can also have the 15q11.2/BP1-BP2 microdeletion referred as the Burnside-Butler susceptibility locus. The BP1-BP2 region spans approximately 500 kb and contains four highly evolutionarily conserved and non-imprinted genes, *NIPA1*, *NIPA2*, *CYFIP1,* and *TUBGCP5,* as shown in Figure 2. BP1-BP2 CNVs have an overall prevalence of 0.5%–1%. Despite deletions and duplications of the locus being equally common, deletions have a more severe clinical impact (Crawford et al., 2019; Burnside et al., 2011). Deletions present with a wide array of clinical symptoms including neurodevelopmental problems, cognitive defects, dysmorphic features, CHD and other abnormalities, but can also display no clinical manifestations. The presentation of the 15q11.2 microdeletion syndrome indicates a low penetrance for abnormal phenotypes caused by the deletion (Mohan et al., 2019; Butler, 2017; Cox and Butler, 2015).

The *non-imprinted in Prader-Willi/Angelman syndrome 1* (*NIPA1)* gene has been associated with autosomal dominant hereditary spastic paraplegia 6 (HSP), a progressive neurodegenerative disorder. *NIPA1* codes for the NIPA1 transmembrane protein and plays a potential role in nervous system development and maintenance. NIPA1 is localised in early endosomes which are recruited to the cell membrane as a response to low levels of extracellular magnesium. The protein is highly expressed in the brain and acts primarily as a Mg^2+^ transporter, but it can also transport other cations such as Fe^2+,^ Mn^2+^, Sr^2+^, and Co^2+^ to a lesser extent (Cox and Butler, 2015; Rafi and Butler, 2020). Based on molecular size and its function, it was inferred that NIPA1 most likely forms a dimer which is required for its activity (Goytain et al., 2007). Mouse *NIPA1* mutants mimic the human HSP phenotype. Additionally, the NIPA1 protein inhibits BMP signalling through targeted degradation of the BMP receptor type 2 (Rafi and Butler, 2020; Wang et al., 2007).

The *non-imprinted in Prader-Willi/Angelman syndrome 2 (NIPA2)* gene is highly conserved and highly expressed in B-lymphocytes and the placenta. The NIPA2 protein has a similar structure and function to NIPA1, being a multi-pass transmembrane protein recruited in response to low extracellular magnesium where it acts as a selective Mg^2+^ transporter (Rafi and Butler, 2020). NIPA2 has been linked with PWS and AS, as well as epilepsy, childhood absence epilepsy, childhood electroclinical syndrome and possibly autosomal recessive congenital ichthyosis. Unlike for *NIPA1*, no mouse model has been made using *NIPA2* mutations or whole gene knock out (KO) (Rafi and Butler, 2020; Jiang et al., 2012; Hildebrand et al., 2014; Bittel and Butler, 2005).

The *cytoplasmic FMR1 interacting protein 1* (*CYFIP1)* gene encodes the CYFIP1 protein which is enriched in membrane ruffles and lamellipodia. The protein is a component of the Wiskott–Aldrich syndrome protein-family verprolin-homologous protein (WAVE1) complex which regulates actin polymerisation at synapses. The gene is expressed widely in all tissues and was shown to be necessary for neuronal and bristle development in *Drosophila* (Rafi and Butler, 2020; Bogdan et al., 2004). It has been linked with MAP kinase signal transduction directing cell growth, survival, and differentiation as well as protein breakdown and mediation of translational repression which impacts learning and memory. CYFIP1 is used by the fragile X syndrome protein (FMRP), encoded by *FMR1,* to repress activity-dependent translation and has thus been linked with fragile X syndrome. Additionally, *CYFIP1* has been associated with autism spectrum disorder (Asd) and an increased risk of schizophrenia and epilepsy. As yet, there are no mouse models (Rafi and Butler, 2020; Napoli et al., 2008; Butler et al., 2022).

The *tubulin gamma complex associated protein 5* (*TUBGCP5)* gene encodes the TUBGCP5 protein which is part of the gamma-tubulin complex required for microtubule nucleation at the centrosome. *TUBGCP5* is widely expressed, with moderate levels in the brain and high levels in the heart and skeletal muscle. The gene has been associated with PWS as well as obsessive compulsive disorder (OCD) and attention deficit disorder (ADD) and was further predicted to be associated with Asd, schizophrenia and other neurodevelopmental disorders. TUBGCP5 has not been studied comprehensively and there are no mouse models (Rafi and Butler, 2020; De Wolf et al., 2013).

### The BP1-BP2 deletion phenotype

Individuals with the 15q11.2 microdeletion display a high degree of phenotypic heterogeneity and low penetrance. These aspects of the deletion make it hard to predict neonatal phenotypes which can range from apparently unaffected to severe. In public databases, such as ClinVar and ClinGen, the BP1-BP2 deletion is considered a recurrent susceptibility CNV. The inheritance pattern for the microdeletion varies between different study cohorts. Where parental data is available, approximately 50%–80% of individuals inherit the BP1-BP2 deletion from an apparently healthy parent, while approximately 15%–30% inherit the deletion from an affected parent. The *de novo* occurrence of the deletion ranges in frequency between 5% and 20% (Mohan et al., 2019; Gardiner et al., 2015; Hashemi et al., 2015; Kang et al., 2021; Rosenfeld et al., 2013; Chu et al., 2021; Benitez-Burraco et al., 2017).

The clinical symptoms of the BP1-BP2 microdeletion syndrome have been grouped into five main subcategories: (1) growth and development, (2) dysmorphic features, (3) intelligence and academic achievement, (4) behavioural and psychiatric problems, and (5) other related medical concerns (Butler, 2017; Cox and Butler, 2015). Although this review will briefly cover other aspects of the syndrome, we will focus on the CHD presentations which have not been highlighted in such a fashion before.

One of the primary characteristics of the BP1-BP2 microdeletion syndrome relates to general developmental and motor delay, which is often accompanied by language delays and speech impediments. Speech delay is particularly widespread, as it was observed in almost all individuals old enough to develop speech at the time of the study (Burnside et al., 2011; Baldwin et al., 2021; Han and Park, 2021; Davis et al., 2019).

Individuals with the 15q11.2 microdeletion do not display a typical craniofacial phenotype–commonly it is described as “dysmorphic features” in the literature – which is present in around 40%–50% of cohorts. Multiple studies have described defects affecting a multitude of features including the shape of the face and cranium overall, ear and nose malformations, as well as palate defects (Burnside et al., 2011; Baldwin et al., 2021; Han and Park, 2021; Davis et al., 2019).

Intelligence and academic achievement markers are difficult to define and assess in patients, particularly young ones. Multiple studies focused on classic intelligence and learning markers which include writing and reading comprehension, memory and IQ tests. These studies reported lower scores on memory tests, reading and writing difficulties caused by dyslexia and dyspraxia, difficulties in rapid adaptation in various tests, lower higher education attendance and lower IQ with frequencies up to 30% (Baldwin et al., 2021; Han and Park, 2021; Davis et al., 2019; Silva et al., 2021; van der Meer et al., 2020; Jonch et al., 2019; Williams et al., 2020).

The microdeletion has also been linked with numerous behavioural and psychiatric problems, with an overall frequency of 70% across various cohorts. Some of the associated conditions include autism spectrum disorder (Asd), Asperger’s, schizophrenia, post-traumatic stress disorder (PTSD), attention deficit disorder (ADD), OCD, self-injurious behaviours, violent tantrums, and paranoid psychosis (Burnside et al., 2011; Benitez-Burraco et al., 2017; Baldwin et al., 2021; Han and Park, 2021; Davis et al., 2019; Silva et al., 2021; van der Meer et al., 2020; Jonch et al., 2019; Williams et al., 2020; Maihofer et al., 2022; Saxena et al., 2019).

Patients with the BP1-BP2 microdeletion also present with other medical concerns which are less frequent and as a result less investigated. Such medical conditions comprise abnormal brain imaging with corresponding clinical seizures and sleeping dysregulation, CHD, genital abnormalities, recurrent infections, and cataracts. The frequencies of such clinical problems vary across cohort studies and display phenotypic heterogeneity. In the case of CHD, approximately 30% of patients across various studies are affected. The abnormalities presented ranges from mild, such as ventricular septal defect (VSD) and atrial septal defect (ASD), to severe malformations such as TOF (Burnside et al., 2011; Baldwin et al., 2021; Han and Park, 2021; Davis et al., 2019; van der Meer et al., 2020; Jonch et al., 2019; Williams et al., 2020; Monteiro et al., 2017).

### BP1-BP2 syndrome and CHD

CHD has been described across many of the BP1-BP2 microdeletion studies, but it is not part of the defining characteristics for the 15q11.2 syndrome, likely due to its low penetrance and phenotypic heterogeneity. The BP1-BP2 microdeletion syndrome was reported to be associated with CHD as early as 2009 (Doornbos et al., 2009), but CHD occurrence may be underestimated in patients partly due to the characteristics of the deletion and partly due to the lack of cardiac monitoring following diagnosis. The omission of investigation and monitoring for CHD is particularly relevant in earlier studies because the primary focus regarding the BP1-BP2 syndrome was developmental delays, neurodevelopmental defects, behavioural issues, and dysmorphic features.

A cohort study in 2012, which characterised the contribution of rare CNVs in the risk of CHD, identified twelve patients with 15q11.2 deletions, at a frequency of 0.53%, as compared to only one individual with BP1-BP2 deletion in the healthy control cohort. Thus, 15q11.2 deletions were more frequent in CHD patients than in controls, indicating a potential association between the two. The participants presented with various cardiac phenotypes which included complex left-sided malformations in three patients (n = 3), coarctation of the aorta (CoA) (n = 3), atrial septal defects (n = 2), ventricular septal defects (n = 2), tetralogy of Fallot (n = 1) and total anomalous pulmonary venous drainage (TAPVD) (n = 1) (Soemedi et al., 2012).

These finding were replicated by later studies. Hashemi et al. (2015) looked at a paediatric cohort and identified the BP1-BP2 deletion with a frequency of 0.76%. The patients had a CHD prevalence of 20% and a wide variety of phenotypes. Some of the cardiac anomalies reported included transposition of the great arteries (n = 1), aortic stenosis (n = 1), ASD (n = 1), and VSD with patent ductus arteriosus (n = 1). A study by Davis et al. (2019), analysed a cohort of patients with the BP1-BP2 deletion to identify differences in clinical features in patients that inherited the deletion. The cohort analysed had a 1.4 male to female ratio and were mostly unrelated. Males were shown to have a statistically significant increase in the clinical and physical phenotypes compared to the females recruited. The cohort revealed an overall prevalence for CHD of 11%.

Chu et al. (2021), described a prevalence of 0.7% of the BP1-BP2 deletion in their foetal and perinatal cohort. Where possible the inheritance of the deletion as well as the phenotype of the parents were analysed. CHD was described in 30% of the infants with the deletion and all the infant CHD cases described had secundum-type ASD with mild pulmonary stenosis. Kuroda et al. (Kuroda et al., 2018), reported a case of familial total anomalous pulmonary venous return (TAPVR) in two affected siblings which was inherited from an unaffected father. No secondary CNVs or pathogenic variants were identified to explain the high level of penetrance in those carrying the deletion.

Additionally, other studies focused on prenatal diagnosis and the clinical outcomes of babies with the 15q11.2 microdeletion. In a cohort of pregnant women, the BP1-BP2 microdeletion was present in 0.21% of cases analysed. Abnormal prenatal ultrasounds, which included foetal malformations, increased nuchal translucency and oligohydramnios, were significantly associated with the presence of the 15q11.2 deletion. The prevalence of cardiovascular malformations identified in the ultrasounds was 16.1%, marking it as the most common anomaly type identified in the cohort, mostly presenting as ventricular septal defects (Kang et al., 2021).

A study of a cohort of individuals referred for intellectual deficiency, behaviour issues and multiple congenital abnormalities identified the prevalence of the BP1-BP2 microdeletion in 0.8% of the cohort population. Of the patients with the 15q11.2 deletion, ∼35% presented with CHD. The patients were investigated using echocardiography, with ages varying from birth to late teenage years. The reported CHD phenotypes included VSD (n = 3), defects in the pulmonary circulation (n = 4), TOF (n = 1), ASD (n = 1), coarctation of the aorta (n = 1), and dextrocardia (n = 1) (Vanlerberghe et al., 2015).

The relevance of the BP1-BP2 deletion has been established when considering both rare and severe or more common and milder forms of CHD. Studies in ostensibly healthy populations have shown that there is a low background frequency of the BP1-BP2 deletion, with a consensus estimate of around 0.25%. Between 5% and 20% of BP1-BP2 deletions arise *de novo.* An analysis looking into the UK BioBank (UKBB) cohort confirmed the increased association of CHD with BP1-BP2 deletion (Williams et al., 2020); among patients in UKBB with CHD, the deletion was more frequently present than among controls. Patients with CHD in the UKBB cohort are almost exclusively mild CHD cases, whereas previous investigations of this deletion had been conducted in patients with more severe CHD phenotypes.

### Genetic/genomic counselling

Investigating the genetic causes of CHD is undeniably important for understanding the disease mechanism, dispensing appropriate treatment and offering genetic counselling regarding reoccurrence. Some of the recommended genetic testing for newborns with congenital defects include DNA microarrays and multiplex ligation-dependent probe amplification (MLPA) which can identify chromosomal abnormalities including CNVs (De Wolf et al., 2013; Monteiro et al., 2017; Morris et al., 2022). However, a tight balance between identifying variants of known significance, benign variants and variants of uncertain significance (VUS) must be maintained. This is particularly relevant when considering the implications of reporting any associations between variants and disease and following up with genetic counselling (Monteiro et al., 2017; Riggs et al., 2020).

The 15q11.2 deletion remains classified as a VUS and presents unique challenges when counselling, similar to other genetic syndromes with variable expressivity and low penetrance. One such challenge is the frequent inheritance from an unaffected parent. The association between the syndrome present in the proband and the 15q11.2 deletion is difficult to make when faced with the absence of any of the described phenotypes in the parents. What is more, this characteristic makes future inheritance patterns and effects hard to predict. The 15q11.2 deletion is mainly tested for in patients with neurodevelopmental disorders or syndromic cases of CHD (De Wolf et al., 2013; Gardiner et al., 2015; Morris et al., 2022). Considering its proven association with an increased risk of CHD (Williams et al., 2020; Monteiro et al., 2017), testing for the 15q11.2 deletion in all CHD cases identified, followed by careful genetic counselling, could increase CHD genetic diagnosis.

## Discussion

Despite being the most common type of birth defect, the genetics and the molecular mechanisms underpinning CHD continue to remain hard to untangle. Various genetic mechanisms, including CNVs, have been shown to play a key role amongst the causes for CHD and require further investigation into downstream consequences. Here we reviewed the association of the BP1-BP2 deletion with CHD.

As with many CNVs, the association of 15q11.2 microdeletion with CHD is characterised by incomplete penetrance, and variable phenotypic expression both with respect to the anatomical CHD diagnosis and accompanying neurodevelopmental defects. In most cohorts the prevalence of CHD for individuals with the 15q11.2 microdeletion ranges from 10% to 30%. Indeed, some smaller studies failed to identify the association of BP1-BP2 deletion with CHD, reflecting the challenge of obtaining sufficient statistical power in the face of such variable penetrance and phenotypic heterogeneity (Cox and Butler, 2015). Another interesting observation is that class I PWS and AS do not include CHD, despite deletion of the BP1-BP2 region. This could be due to gene dosage effects, gene-gene interactions or transacting factors, but remains unsolved to date.

The relevance of the BP1-BP2 microdeletion in relation to both rare and common form of CHD has been well established in preexisting data and publications. Due to its characteristics, the deletion remains a VUS presenting a difficult challenge for genetic counsellors, but is undeniably a predisposing factor for CHD and should be carefully tested for and considered by the clinical genetic services (De Wolf et al., 2013; Gardiner et al., 2015; Williams et al., 2020; Monteiro et al., 2017; Riggs et al., 2020). Despite this, studies investigating approaches to reduction of CHD risk in affected families have not yet been possible due to the lack of a mechanistic understanding of the consequences of BP1-BP2 deletion on heart development. We look towards further publications that can shed a light on the intriguing link between the 15q11.2 microdeletion and CHD.
